# Magnetic particle imaging resolution needed for magnetic hyperthermia treatment planning: a sensitivity analysis

**DOI:** 10.3389/fther.2025.1520951

**Published:** 2025-02-16

**Authors:** Shreeniket Pawar, Nageshwar Arepally, Hayden Carlton, Joshua Vanname, Robert Ivkov, Anilchandra Attaluri

**Affiliations:** 1 Department of Mechanical Engineering, School of Science, Engineering, and Technology, The Pennsylvania State University Harrisburg, Harrisburg, PA, United States; 2 Department of Radiation Oncology and Molecular Radiation Sciences, The Johns Hopkins University School of Medicine, Baltimore, MD, United States; 3 Department of Oncology, Johns Hopkins University School of Medicine, Baltimore, MD, United States; 4 Department of Mechanical Engineering, Whiting School of Engineering, Johns Hopkins University, Baltimore, MD, United States; 5 Department of Materials Science and Engineering, Whiting School of Engineering, Johns Hopkins University, Baltimore, MD, United States

**Keywords:** magnetic nanoparticle hyperthermia, magnetic particle imaging, magnetic iron oxide nanoparticle, computed tomography, finite element analysis, bioheat transfer simulation, specific loss power

## Abstract

**Purpose:**

Magnetic particle imaging (MPI) is a nascent tracer imaging modality that generates images from magnetic iron oxide nanoparticles (MIONs) in tissue. MPI resolution is a critical input parameter for defining the reliability of simulations-based temperature predictions for magnetic nanoparticle hyperthermia (MNPH). The objective of this study was to ascertain how spatial resolution provided by MPI data affects the reliability of predicted temperatures and thermal dose in simulations using MPI data as inputs.

**Methods:**

Computed tomography (CT) and MPI scans obtained from a tumor injected with MIONs were co-registered to align their coordinates. Co-registered data were used to obtain geometry and volumetric heat sources for computational simulations of MNPH in phantom tumors. In addition to using the MPI-derived *in vivo* MION distribution (D1) we analyzed two mathematical MION distributions: uniform (D2) and Gaussian (D3). All distributions were discretized into cubic voxels and the data were imported into a commercial finite element bioheat transfer (FEBHT) software for thermal simulations. FEBHT simulations were conducted using the Pennes’ bioheat equation using four different MION specific loss power (SLP) values in the range 300–600 [W/g Fe]. The impact on predicted temperature resolution and thermal dose of spatial resolution were assessed by varying the linear voxel density (LVD) from 0.36 to 4.06 [voxel/mm]. Results were compared against the simulation with the highest LVD [4.06(voxel/mm)], where deviations in temperature of ≤ ±1 [°C] and thermal dose coverage ≤ ±5 [%] were deemed acceptable.

**Results:**

The D3 distribution resulted in the highest predicted temperatures, followed by D1 and D2; however, in terms of thermal dose, D1 showed lowest tumor coverage, requiring higher heat output from MIONs than was required for the other distributions studied. The results of the sensitivity analysis revealed that the predicted tumor temperature increased with LVD across all tested SLP values. Additionally, we observed that the minimum acceptable LVD increased with SLP.

**Conclusion:**

Current (preclinical small animal) MPI scanners provide sufficient spatial resolution to predict temperature to within ±1 [°C], and thermal dose coverage to within ±5 [%] for MION formulations having heat output SLP = <370 [W/g Fe]. Higher spatial resolution is needed to achieve a similar precision when MION SLP exceeds 370 [W/g Fe]. We also conclude from the results that assuming a uniform MION distribution in tissue, which has been a common practice in MNPH simulations, overestimates the SLP needed to deposit meaningful thermal dose.

## 1 Introduction

Magnetic nanoparticle hyperthermia (MNPH) is a therapy that can deliver heat energy precisely to cancer tumors (Gilchrist et al., 1957). MNPH involves administering magnetic iron oxide nanoparticles (MIONs) directly to a tumor and heating them by exposing the region to an alternating magnetic field (AMF) (Gilchrist et al., 1957; Rosensweig, 2002). AMF with a frequency in the low radiofrequency range of about 100–300 [kHz], penetrates tissue with little attenuation, making it particularly effective for deep-seated tumors (Hensley et al., 2017; Rodrigues et al., 2017). Challenges arise from inconsistent or unreliable MION concentrations in the target after delivery, and their typically heterogeneous distributions within tissue, necessitating the development of imaging-guided treatment plans to ensure adequate thermal dose in tumors while minimizing damage to surrounding healthy tissues (Rodrigues et al., 2017; Kut et al., 2012; Reis et al., 2016; Suleman and Riaz, 2020a).

Magnetic particle imaging (MPI) is a nascent tracer imaging modality that provides particle specific imaging, and thus presents opportunities to address limitations in energy delivery and control during MNPH (Healy et al., 2022). As with other tracer imaging modalities, MPI data require co-registration with magnetic resonance imaging (MRI), computed tomography (CT), or other anatomical imaging modality to enable clinical diagnostic and therapeutic application. While spatial resolution often directly dictates clinical utility for diagnostic imaging, its effect on MNPH treatment planning may be indirect, and requirements for guiding MNPH may even differ from those required for diagnostic imaging. Thus, if MPI data are to be used as inputs for thermal simulations, the effects of spatial resolution of imaging data on accuracy and precision of predicted outcomes need to be understood to ascertain reliability of simulations outcomes. Specifically, is resolution of an MPI image a critical factor affecting precision of predicted temperature? If yes, what is the resolution required to predict temperatures within clinical acceptable uncertainty? No studies have explored effects of MPI spatial resolution on precision of simulated tissue temperature rise caused by heating MIONs in tissues.

Current MNPH treatments involve closed-loop control systems to monitor and adjust MION heat output with little information about their distribution within the tumor (Xiaohua et al., 2015; Ahmed et al., 2022; Sharma et al., 2023). Temperature probes, typically constructed from optical fibers, are inserted into the tumor to monitor tissue temperature during treatment. Often, only one probe is used, and treatment is conducted with plans using only basic principles. Energy delivery is controlled manually to achieve a minimum target temperature, as measured by the probe, with the thermal dose estimated during post-treatment analysis. This is in sharp contrast to the workflow used in other energy-based interventions, such as radiation therapy, which begins with the development of a prescriptive treatment plan using simulations-based algorithms for which the processed anatomical imaging data defining the tumor volume are inputs. Thus, by comparison current MNPH workflows are rudimentary, and often fail to meet most clinical quality assurance guidelines (Healy et al., 2022).

A critical barrier to developing robust treatment plans for MNPH is the lack of clinical imaging tools that unambiguously identify MION content and distribution in tissue. Computational models, crucial for refining MNPH, have progressed, but still lack reliable input from imaging. Homogeneous or mathematical MION distributions are often assumed in order to reduce computational burden (Rodrigues et al., 2017; Pennes, 1948; Kamal Kandala et al., 2019; Panagiotopoulos et al., 2015). Consequently, simplified mathematical assumptions of the MION distribution(s) used in simulations often produce treatment plans that fail to predict accurately tissue temperatures realized during MNPH. Indeed, assessing reliability for quality assurance using this approach is uncertain because of a lack of critical pre-treatment data (Kamal Kandala et al., 2019; Panagiotopoulos et al., 2015). MPI can overcome these limitations; however, a critical question to be addressed is the spatial resolution required as input data to ensure reliable tissue temperature predictions for MNPH treatment plans (Panagiotopoulos et al., 2015; Borgert et al., 2012; Tay et al., 2018; Gleich and Weizenecker, 2005; Goodwill and Conolly, 2010).

MPI measures the magnetic moment of a sample containing magnetic materials, typically MIONs generates a linear signal, enabling the detection of trace amounts of MIONs [<1 × 10^−8^ (g)]. Actual detection limits however, depend on the magnetic properties of the MIONs and scanner capabilities (Gleich and Weizenecker, 2005; Goodwill and Conolly, 2010). Tissue, being diamagnetic, generates no signal; thus, MPI requires co-registration with other imaging modalities, such as CT, to identify anatomical features. When combined with anatomical images, MPI provides the capability to image the MION distribution in a tumor and to quantify the MION concentration. Preliminary studies indicate that integrating MPI with CT enables quantification of MION concentration, establishing a linear correlation between MPI pixel density, local MION concentration, and MION volumetric heat generation at a constant magnetic field (Carlton et al., 2024a; Koch and Winfrey, 2014).

The spatial resolution of MION deposits in MPI images depends on both the MION magnetization and scanner properties (Panagiotopoulos et al., 2015; Borgert et al., 2012; Tay et al., 2018; Gleich and Weizenecker, 2005; Goodwill and Conolly, 2010). Current small animal preclinical scanners enable MION detection in the nanogram range [~1 × 10^−8^ (g MION)] with submillimeter voxel resolution [~0.25 (mm/voxel)]; however, specifically for MNPH, the encountered challenges arise from high MION concentrations [>1 × 10^−2^ (g MION/g tissue)], which produce high signal intensity (brightness) that, at times, can saturate detectors and yield spurious false or “ghost” images or “halos” in regions containing no MIONs (Paysen et al., 2019). Scanner adjustments to minimize and correct these effects are necessary to image MIONs at such high concentrations; however, these adjustments can also reduce the image resolution and quantitation of MIONs in regions containing lower concentrations (Carlton et al., 2024a).

Accepting that MPI-guided MNPH treatment planning simulations will improve treatment reliability raises another question: How much resolution or precision is required? Increasing the spatial resolution and anatomic detail inevitably raises the demand for computational resources. Increased spatial resolution would likely increase scan time, data storage requirements, and time to develop and approve treatment plans based on much larger imaging data files generated by high-resolution (three-dimensional) scans. At some point, the increased demand for time to develop treatment plans and computing resources (including data storage) becomes prohibitive, impractical, expensive, or impedes timely execution of quality patient care. Moreover, tissue thermal transport properties are coupled with physiological heat-induced responses in vascular perfusion and tissue fluid reservoirs. These factors also influence the resolution required to achieve reliable model predictions for patient care.

Our objective here was to ascertain how MION heat output and MPI scanner spatial resolution affect the reliability of calculated tissue temperature and estimated thermal dose [cumulative equivalent minutes at 43 (°C) (CEM43) >15 (min)]. For the simulations, we assumed a range of specific loss power (SLP) values between 300–600 [W/g], which have been previously reported with demonstrated utility to treat solid tumors in animal models (Carlton et al., 2024a; Carlton and Ivkov, 2023; Carlton et al., 2023b; Ota et al., 2021; Carlton et al., 2024a). For simplification of data representation, we used isotropic voxel dimensions represented as linear voxel density (LVD, i.e., density of voxels per unit side length) from 0.36 to 4.06 [voxel/mm]. Maximum and minimum LVD values were chosen based on the highest resolution of small animal MPI and the expected highest resolution of human-scale MPI scanners (Tay et al., 2019; Graeser et al., 2019). Using these SLPs and LVDs, we performed a computational sensitivity analysis to identify the minimum needed to maintain simulation-based precision of temperature and thermal dose coverage to within ±1 [°C] and ±5 [%], respectively.

## 2 Materials and methods

### 2.1 MPI and CT equipment

MPI images were obtained using a Momentum^©^ MPI (Magnetic Insight, Inc., Alameda, CA) scanner operating at a drive frequency of 45 [kHz]. Cone Beam CT (CBCT) images were acquired using a Small Animal Radiation Research Platform (SARRP), Xstrahl Inc. Suwanee, GA) (Wong et al., 2008).

### 2.2 MION calibration

Synomag^®^-D 70 [nm] magnetic iron oxide nanoparticles (micromod Partikeltechnologie GmbH, Lot#: 09122104–02) suspended in water were purchased and used at a concentration of 50 [mg Fe/mL]. The concentration was verified using a ferene-s assay (Hedayati et al., 2018). We digested a small aliquot of the particles [~1 (μg Fe)] in a solution of ascorbic acid and acetate buffer for 20 [h] before measuring the concentration. Using a calibration curve of iron standard solutions as reference, we then calculated the concentration of iron in the solution. To correlate MPI signal with thermal output, we imaged a concentration gradient consisting of four particle samples with increasing concentration: 0.1, 0.5, 1.0, and 5.0 [mg Fe/mL]. We plotted the mean voxel intensity value within each calibration sample against the particle concentration.

### 2.3 *In vivo* imaging data acquisition

#### 2.3.1 Mouse model and tumor implantation

The Johns Hopkins Institutional Animal Care and Use Committee approved all animal studies. A single 10-week-old female BALB/c mouse (Jackson Laboratory, Bar Harbor, ME) was used for the study. The mouse was fed a normal diet and water *ad libitum* and maintained on a 12-h light/12-h dark cycle, and we examined the mouse daily for signs of distress or pain. In previous work, we analyzed the intratumor heating of this mouse (Carlton et al., 2023a).

We used the murine mammary carcinoma cell line 4T1 [ER/PR/HER2 negative], which was purchased from the American Type Culture Collection (ATCC; Manassas, VA, United States). The cells were grown in Roswell Park Memorial Institute (RPMI) 1,640 medium, which contained 10 [%] heat-inactivated fetal bovine serum (FBS). We then performed a subcutaneous injection of 2.5 × 10^5^ cells suspended in sterile PBS [50 (μL)] into the right thigh of the mouse. Afterwards, we monitored tumor growth three times per week with calipers.

#### 2.3.2 MION injection

Upon reaching a volume of 100–400 [mm^3^], the mouse was anesthetized by inhalation of 1–2 [%] isoflurane with O_2_. Once anesthetized, 11 [μL] of Synomag^®^-D 70 nm MIONs suspended in sterile water at a concentration of 25 [mg Fe/mL H_2_O] was injected into the tumor. The nanoparticle injection was performed using a syringe pump (Pump 11 Elite, Harvard Apparatus, Holliston, MA) set to a rate of 2.5 [μL/min].

#### 2.3.3 *In vivo* imaging

The *in vivo* 3D MPI scan consisted of 21 isotropic radial slices with a gradient field of 5.7 [T/m] and 5 [mT] excitation field. For imaging, we anesthetized the mouse with 1–2 [%] isoflurane; respiration rate was monitored using a sensor on the chest (Small Animal Instruments, Stony Brook, NY, United States) kept between 50–80 [breaths/min]. Mouse core body temperature was monitored rectally with a fiber optic probe (Small Animal Instruments, Stony Brook, NY, United States) and a custom holder linked to a circulating heated water bath was used to maintain core body temperature at approximately 37 [°C]. CBCT images [60 (kVp) at 0.8 (mA) current for 60 (s)] were gathered using the same configuration as the MPI scans, where 230 projections were recorded.

#### 2.3.4 Image co-registration and MPI data extraction

Data acquired from the Momentum^©^ MPI and CBCT scanners have distinct imaging centers of origin, which complicates direct comparisons. MPI specifically generates signal from MIONs, whereas CBCT provides anatomical information. To facilitate co-registering the two sets of imaging data, fiducials were manually placed and aligned for both the MPI and CBCT images (Carlton et al., 2024a). The fiducials consisted of ~1 [μL] aliquots of VivoTrax (Magnetic Insight, Inc., Alameda, CA) placed and fixed at arbitrary locations within the MPI field of view. The fiducials also provided sufficient contrast for CBCT scans. Co-registration and data extraction were performed using Materialize Mimics^©^ Research v.25 (Materialise NV, Leuven, Belgium), where both MPI intensity values (recorded in grayscale) and voxel coordinates were exported as a text file. The bulk of the workflow used in this study was previously developed and described (Carlton et al., 2024a). For this study, we plotted the data as a point cloud in MATLAB^®^, with each point representing a specific grayscale value at a unique coordinate. The gray values were then converted into arbitrary units [a.u.] by assuming a linear relationship between grayscale values and [a.u.]. These [a.u.] values were further transformed into a volumetric heat source [W/m^3^] using a calibration curve obtained from the experimental data and imported into the commercial FEA software COMSOL Multiphysics^®^ for heat transfer simulations.

### 2.4 MION distributions and voxelization

**FIGURE 1:**
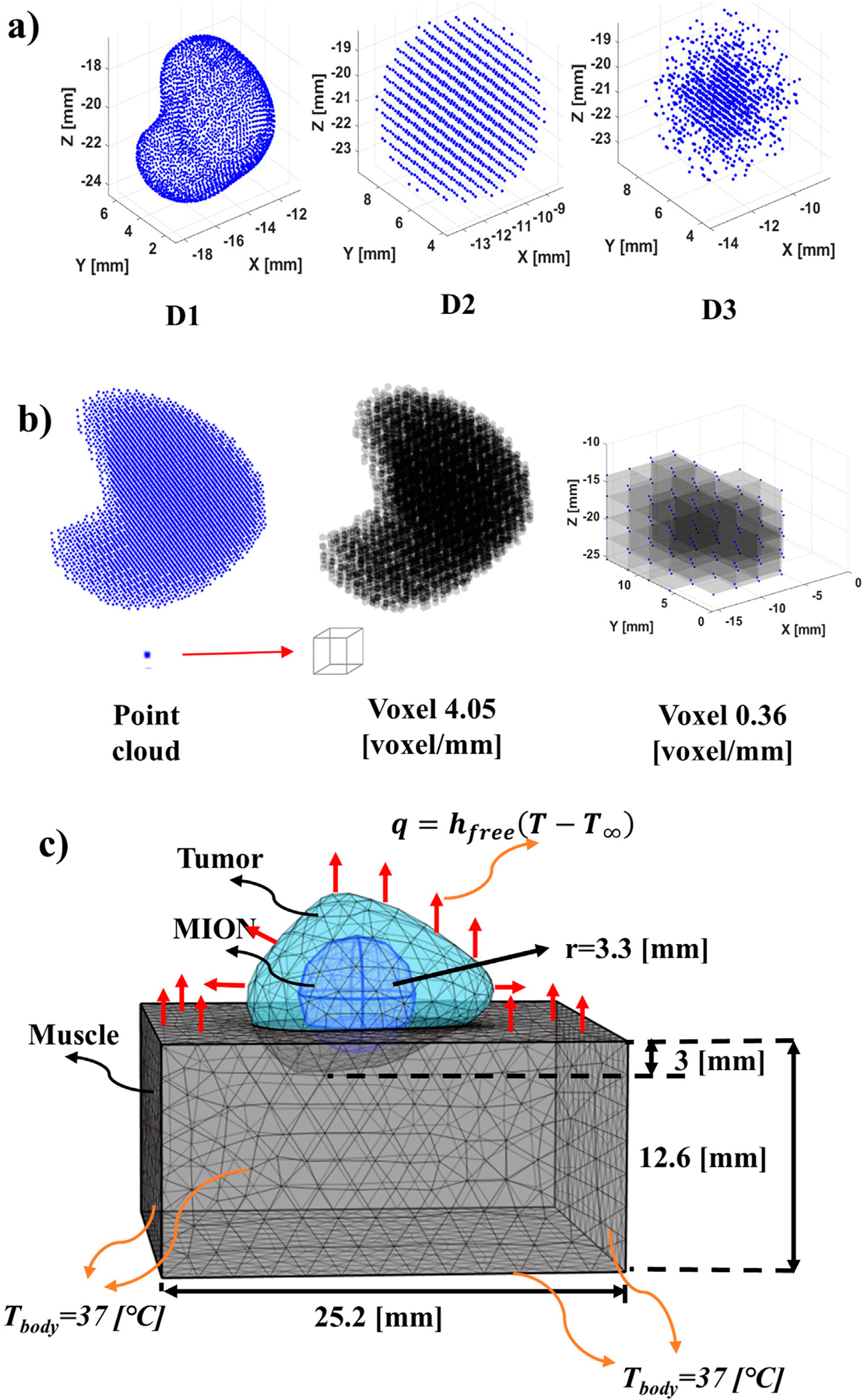
Schematic of phantom MION heat source, geometry and boundary conditions. **(A)** Distribution of MION for the study: D1 (*in vivo*) obtained from the co-registration of MR/CT and MPI images. D2 (uniform) is obtained from the conservation of mass and volume of the MION from the D1 and distribution of the MION with a constant density over the volume of the tumor phantom. D3 (Gaussian) is obtained from the conservation of mass and volume of the MION from the D1 and distributed the MION in the form of a D3. **(B)** Conversion from point cloud (array) to voxel (3D volume) of a MPI image at different resolutions. The point cloud of pixel intensity obtained from an MPI image was converted into a voxel by assuming that each point in the voxel was the corner of the cuboid. Formation with the voxel of 4.06 [voxel/mm] [66.92 (voxels/mm^3^)] resolution from the point cloud. To decrease the resolution of the obtained voxel, the average of the voxel was taken, that is, eight smaller voxels formed one larger voxel. A voxel of 0.36 [voxel/mm] [0.05 (voxel/mm^3^)] feature size was formed through the subsequent addition of the voxel to form a larger voxel. **(C)** Geometry and boundary conditions for the FEBHT software with muscle, tumor and MION. The MION for the D1 was obtained from segmentation of MPI data. The surface of the muscle inside the body was assumed to be 37 [°C] and the outer surface was assumed to have free convection heat transfer to the surroundings.

In addition to information of MION distribution gathered from MPI (D1), two additional mathematically generated MION distributions were analyzed: a uniform distribution (D2), and a Gaussian distribution (D3); both having the center of the phantom tumor being the coordinate origin (Figure 1A). In the case of D2 and D3, the MION mass and volume from D1 was conserved. The D2 distribution assumed a uniform MION distribution throughout the spherical (phantom tumor) volume. The D3 distribution was modeled using a Gaussian probability function P(x,y,z) (Kamal Kandala et al., 2019; Kreyszig, 1983; Suleman and Riaz, 2020b), using the center of a sphere (phantom tumor) as the origin of the MION mass, with MION concentration decreasing to the boundary. We assumed spherical symmetry, or a consistent spread of nanoparticles in all directions, by fixing variances of nanoparticle content in x,y, and z dimensions to be equal to a single standard deviation, $\sigma_{x}=\sigma_{y}=\sigma_{z}$.

(1) $$P(x,y,z)=\frac{1}{(2\pi {)}^{3/2}\sigma_{x}\sigma_{y}\sigma_{z}}e^{\left(-\frac{1}{2}\left(\frac{x^{2}}{{\sigma_{x}}^{2}}+\frac{y^{2}}{{\sigma_{y}}^{2}}+\frac{z^{2}}{{\sigma_{z}}^{2}}\right)\right)}$$

The total deposited heating rate, $Q_{\mathrm{heat}}[W]$, was kept constant among the models.

A MATLAB^®^ script was used to discretize the point cloud distributions. Our script represented the data as a spatially discretized point cloud, and each point was converted into a cubic voxel (Roberson, 2021). To reduce the number of parameters for visualization we used LVD of cuboid with equal sides, which is common practice in computational imaging. This simplification facilitates data representation of complex shapes. To reduce the LVD, the script spatially averaged the MPI grayscale values around each point. The voxel size range we used for sensitivity analysis was varied from 0.25 [mm/voxel side length] [0.01 (mm^3^/voxel)] (resolution from the MPI DICOM files) to 2.78 [mm/voxel side length] [21.48 (mm^3^/voxel)], or LVDs of 4.06 [voxel/mm] [66.92 (voxels/mm^3^)] to 0.36 [voxel/mm] [0.05 (voxel/mm^3^)], respectively.

### 2.5 FEA

#### 2.5.1 Geometry and material properties

The segmented MION and tumor domains from the co-registered MPI and CBCT images were imported into COMSOL Multiphysics 6.2 (Carlton et al., 2024a). For D1, the segmented MPI data were used to represent the MION domain. However, for D2 and D3 distributions, the spherical geometry of the MION domain was assumed to have total volume equal to that used for the D1 MION domain. The phantom subcutaneous tumor was partially embedded in a cuboid for which we assumed physical and thermal transport properties of the surrounding tissue phantom (cuboid) to be equivalent to that of idealized muscle (Banura et al., 2016). The details of the geometry, solver parameters, and phantom tumor model with MION distributions are summarized in Tables 1, 2, and represented schematically in Figure 1C, respectively.

#### 2.5.2 Governing equations and boundary conditions

To model transient heat transfer from MION heating in the segmented subcutaneous tumor model, Pennes’ bioheat equation (Pennes, 1948; Zhu, 2009) (Equation 2) was used in COMSOL Multiphysics. Pennes bioheat equation assumes that all the domains are solid, allowing the model to solve a single governing equation instead of separate equations for heat conduction, mass conservation, and fluid flow (Navier-Stokes) reducing computational resources. Pennes’ bioheat transfer has been previously verified with no perfusion and validated for thermal therapies (Andrä et al., 1999; Fuentes et al., 2010). However, the model is validated for microvasculature, it is not suitable for domains with large blood vessels. The verification of the current numerical solver is shown in Supplementary Material.

(2) $${\left(\rho c_{p}\right)}_{n}\frac{\partial T_{n}}{\partial t}=\nabla \cdot k_{n}\nabla T_{n}+(1-\epsilon )\omega_{bl,n}{\left(\rho c_{p}\right)}_{bl}\left(T_{bl}-T_{n}\right)+Q_{m,n}+Q_{P}$$

Here, bl and n represent blood and tissue (tumor, n=1; muscle tissue, n=2), respectively. $\rho_{bl},c_{bl},\omega_{bl,n}$, and $T_{bl}$ denote density, specific heat, perfusion rate, and temperature, respectively. $\rho_{n},c_{n},k_{n},\;T_{n},Q_{m,n}$ denote the density, specific heat, thermal conductivity, local temperature, and metabolic heat generation rate, respectively, for either the tumor or healthy tissue, and t is heating time. The adjustable equilibration parameter ϵ (ranging from zero to one) was assumed to be uniform throughout the tissues ϵ=0 in this study, where $Q_{P}$ represents the volumetric heat generated by the MION domain.

To account for the effects of temperature on blood perfusion, a modified Arrhenius perfusion (Kamal Kandala et al., 2019; He et al., 2004; Schutt and Haemmerich, 2008) was considered (Equations 3, 4).

(3) $$\omega_{bl}(T)=\left\{\begin{array}{l} \omega_{bi}(30\times DS+1),(DS\leq 0.02)\\ \omega_{bi}(-13\times DS+1.86),(0.02<DS\leq 0.08)\\ \omega_{bi}(-0.79\times DS+0.884),(0.08<DS\leq 0.97)\\ \omega_{bi}(-3.87\times DS+3.87),(0.97<DS\leq 1.0) \end{array}\right.$$

(4) $$DS=1-e^{-\left(A\int_{0}^{t}e^{-\frac{E_{a}}{RT(\tau )}}d\tau \right)}$$

Here, $\omega_{b_{i}}$ is the constant perfusion value [1/s] and DS is the degree of vascular stasis. A represents the frequency or pre-exponential factor [1/s], R is the universal gas constant $]\frac{J}{\;\mathrm{K.mol}\;}],\;T(\tau )$ is absolute tissue temperature as a function of time, and $E_{a}$ is activation energy $\left[\frac{J}{mol}\right]$ as expressed in Equation 3.

Free-convection boundary conditions (Equation 5) were assumed at all the outside boundaries of the tumor and muscle tissue domains (Nellis and Klein, 2008):

(5) $$q^{\prime \prime }=h_{\mathrm{free}}\left(T-T_{\infty }\right)$$

The ambient temperature, $T_{\infty }$, and free convection heat transfer coefficient, $h_{\mathrm{free}}$, were set to 20 [°C] and 3.7 [W/(m^2^·K)], respectively (Banura et al., 2016). Continuity of the temperature and heat flux at the domain interfaces was assumed. Muscle tissue boundaries were distant from the tumor, that is, in contact with the rest of the body, and the initial tissue and tumor temperatures were set to a temperature, $T_{\mathrm{body}}$, of 37 [°C] (Figure 1C). The simulation was run for 21 [min] and was followed by a 4-min cooling period.

The SLP range for this study was based on values obtained from a review of literature for Synomag D70 (Carlton et al., 2024a; Carlton and Ivkov, 2023; Carlton et al., 2023b; Ota et al., 2021). We selected four increasing SLP values, 300, 400, 500, and 600 [W/g Fe], within the range of reported values in order to determine the effect of SLP on the intratumor temperature and thermal dose coverage.

The volumetric heat source used in this study is shown in Equation 6:

(6) $$Q_{P}=Q_{P,i}\times P$$

where $Q_{P}\left[W\times m^{-3}\right]$ is the volumetric heat source, i represents the three distributions D1, D2, and D3, respectively, and P is a rectangular pulse of 60 [s] ON with 10 [s] OFF. The significance of pulsed AMF in minimizing eddy current heating has been previously described (Attaluri et al., 2020; Ivkov et al., 2005).

MION volume and heat input were conserved. The evaluation of the heat source for D1 was obtained from the co-registered MR/CT and MPI images. The volumetric heat source for D2 was obtained using Equation 7:

(7) $$Q_{P,\mathrm{uniform}}=\frac{{∭}_{\Omega }Q_{P,\mathrm{in}\;\mathrm{vivo}\;}dV}{V_{MION}}$$

where $Q_{P,\mathrm{uniform}}\left[W\times m^{-3}\right]$ is the uniform volumetric heat source; $Q_{P,\mathrm{in}\;\mathrm{vivo}}\left[W\times m^{-3}\right]$ is the D1 volumetric heat source; Ω is the domain of the MION obtained from the co-registered data; $dV\;\left[m^{3}\right]$ is the infinitesimal volume element of the MION distribution; and $V_{\mathrm{MION}}\left[m^{3}\right]$ is the total volume of MION mass for a uniform distribution.

D3 is a Gaussian volumetric heat source that can be represented as Equation 8 obtained from Equation 1,

(8) $$Q_{P,\mathrm{gaussian}\;}=Q_{P,\mathrm{gaussian}\text{,}max}\times \frac{1}{(2\pi {)}^{3/2}\sigma_{x}\sigma_{y}\sigma_{z}}\times e^{\left(-\frac{1}{2}\left(\frac{{\left(x-\mu_{x}\right)}^{2}}{\sigma_{x}^{2}}+\frac{{\left(y-\mu_{y}\right)}^{2}}{\sigma_{y}^{2}}+\frac{{\left(z-\mu_{z}\right)}^{2}}{\sigma_{z}^{2}}\right)\right)}$$

where $Q_{P,\mathrm{gaussian}\;}\left[W\times m^{-3}\right]$ is the Gaussian volumetric heat source; $Q_{P,\mathrm{gaussian}\text{,}max}[W]$ is the peak value of the heat source in the D3; $\mu_{x}\;[mm],{\;\mu }_{y}[mm]$ and $\mu_{z}[mm]$ are the mean positions of the D3 distribution in x-,y-, and z-axes; and $\sigma_{x}[mm],\sigma_{y}[mm]$ *and* $\sigma_{z}[mm]$ are the standard deviations of the distribution, which was assumed to be constant σ [mm].

The conservation of energy in distributions D2 and D3 is given by Equation 9:

(9) $${∭}_{\Omega }Q_{P,\mathrm{gaussian}}dV={∭}_{\Omega }Q_{P,\mathrm{uniform}}dV$$

D3 can be approximated three times the standard deviation of the mean, as shown in Equation 10. All heat was assumed to be distributed in the volume occupied by the MIONs, therefore, $3\times \sigma =r_{\mathrm{MION,gaussian}}$.

(10) $$\overset{\mu +3\sigma }{\underset{\mu -3\sigma }{\int \int \int }}Q_{P,gaussian}dV≅\overset{\infty }{\underset{-\infty }{\int \int \int }}Q_{P,gaussian}dV$$

Substituting Equation 9 into Equation 10, we obtain Equation 11.

(11) $$Q_{P,gaussian,\max\;}=\frac{Q_{P,uniform\;}\;\times \;V_{MION}\;\times \;{\left(2\pi \right)}^{3/2}\sigma^{3}}{{\int \int \int }_{\mu -3\sigma }^{\mu +3\sigma }e^{\left(-\frac{1}{2}\left(\frac{{\left(x-\mu_{x}\right)}^{2}+{\left(y-\mu_{y}\right)}^{2}+{\left(z-\mu_{z}\right)}^{2}}{\sigma^{2}}\right)\right)}dx\;dy\;dz}$$

Integrating Equation 11 and substituting the values from Table 1, we obtain Equation 12.

(12) $$Q_{\mathrm{P,gaussian,max}}=7.181\times Q_{P,\mathrm{uniform}\;}$$

Therefore, D3 was modeled as shown in Equation 13.

(13) $$Q_{P,\mathrm{gaussian}\;}=Q_{\mathrm{P,gaussian,max}\;}\times \frac{e^{-\frac{1}{2}\frac{{\left(x-\mu_{x}\right)}^{2}}{\sigma^{2}}}}{{\left(2\pi \right)}^{\frac{1}{2}\sigma }\sigma }\times \frac{e^{-\frac{{\left(y-\mu_{y}\right)}^{2}}{\sigma^{2}}}}{{\left(2\pi \right)}^{1/2}\sigma }\times \frac{e^{-\frac{1}{2}\frac{{\left(z-\mu_{z}\right)}^{2}}{\sigma^{2}}}}{{\left(2\pi \right)}^{1/2}\sigma }$$

### 2.6 Data and sensitivity analysis

#### 2.6.1 Thermal analysis

The temperature and CEM43 obtained from FEBHT simulations were processed for all combinations of LVD and SLP values. Three key metrics were analyzed: (1) the spatiotemporally averaged temperature rise, denoted as the *average* ΔT; (2) the temporal average of the spatial maximum temperature rise, denoted as the *maximum* ΔT; and (3) the percentage of tumor volume with CEM43 > 15 (min), referred to as *thermal dose coverage*. In this case, ΔT refers to the overall temperature change from the baseline temperature of 37 [°C].

These metrics were selected based on their relevance to energy conservation and treatment precision. The *average* ΔT serves to verify energy conservation across different distributions for a given voxel size. It was anticipated that the *average* ΔT would differ insignificantly among distributions due to non-linear perfusion and uneven convective losses. The *maximum* ΔT was included due to the reliance on point thermometry in current MNPH systems for predicting treatment outcomes. Given the importance of accurately predicting the maximum temperature, this metric was prioritized to enhance the precision of predicted outcomes. Finally, *thermal dose coverage* was selected as a quantitative measure of treatment efficacy, since a thermal dose range of 15–60 (min) is typically used as a clinical thermal dose objective for radiation sensitization (Van Rhoon et al., 2013).

#### 2.6.2 Criteria for sensitivity analysis

For our sensitivity analysis, we measured how the selected metrics (2.6.1) changed when the LVD and SLP values were varied. We first assumed the distribution with the highest LVD (resolution of DICOM files from the MPI scanner) to be the “exact” solution. As we decreased the LVD in subsequent simulations, we recorded the value when simulation temperature exceeded a ±1 [°C] deviation from the exact simulation or a ±5 [%] of the tumor volume with CEM43 > 15 [min]. We denoted this as the minimum acceptable LVD. This was repeated for each of our selected SLP values.

#### 2.6.3 Extrapolating data convergence using logarithmic regression analysis

In addition to determining the minimally acceptable LVD from the simulations, we also analysed data convergence. We used a logarithmic regression to fit the data, as shown in Equation 14:

(14) y=Aln(LVD)+B

where y represents the simulation metric. We extrapolated the logarithmic fit to determine the theoretical LVD value where convergence would occur. We defined the convergence criteria using the simulation results from the lowest SLP condition [300 (W/g Fe)]. Using the log fits for the 300 [W/g Fe] datasets, we calculated the LVD where the *maximum* ΔT and *thermal dose coverage* exceeded ± 1 [°C] and ± 5 [%], respectively, for all the distributions [LVD=2.71(mm/voxel)], which we denoted as $LVD_{\mathrm{sat}}$. We then calculated the convergence slope at that point for each distribution as shown in Equation 15:

(15) $$\frac{dy}{d(LVD{)}_{\mathrm{conv}\;}}=\frac{A_{\mathrm{sat}}}{LVD_{\mathrm{sat}\;}}$$

where $\frac{dy}{d(LVD{)}_{\mathrm{conv}}}$ is the slope of the converged curve from Equation 14 and $A_{\mathrm{sat}\;}$ is the saturated regression coefficient.

The logarithmic function for each SLP was extrapolated until the slope was less than or equal to the convergence slope shown in Equation 16. Using Equations 15, 16 we get Equation 17:

(16) $$\frac{dy}{d(LVD{)}_{i}}\leq \frac{dy}{d(LVD{)}_{conv}}$$

(17) $$LVD_{reqd,i}\geq \frac{LVD_{\mathrm{sat}\;}\times \;A_{i}}{A_{\mathrm{sat}}}$$

where $\frac{dy}{d(LVD{)}_{i}}$ is the slope of the log function of the *i*^th^ SLP [i=400,500 and 600 (W/g Fe)], $LVD_{reqd,i}$ is the required LVD for a given SLP and distribution, and $A_{\text{i}}$ is the regression co-efficient at i^th^ SLP. We recorded the LVD value where $\frac{dy}{d(LVD{)}_{i}}=\frac{dy}{d(LVD{)}_{\mathrm{conv}}}$, denoted as $LVD_{reqd}$. After obtaining $LVD_{reqd}$ values, we plotted them with respect to SLP and used linear and quadratic interpolation to estimate the SLP value corresponding to the MPI scanner resolution, LVD=4.06[voxel/mm], that would be required to achieve those results.

#### 2.6.4 Regression fitting

For all the data a weighted linear regression was performed for data points that increased linearly. For all regression, a goodness of fit lower than 0.95 a quadratic regression was used. It was anticipated that MION concentration will be directly proportional to mean voxel value and volumetric heat source will be directly proportional to mean pixel values. *Maximum* ΔT, *average* ΔT and *thermal dose coverage* were anticipated to converge to a certain value. Therefore, a logarithmic fit was performed on these metrics.

## 3 Results

### 3.1 MPI voxel calibration

**FIGURE 2:**
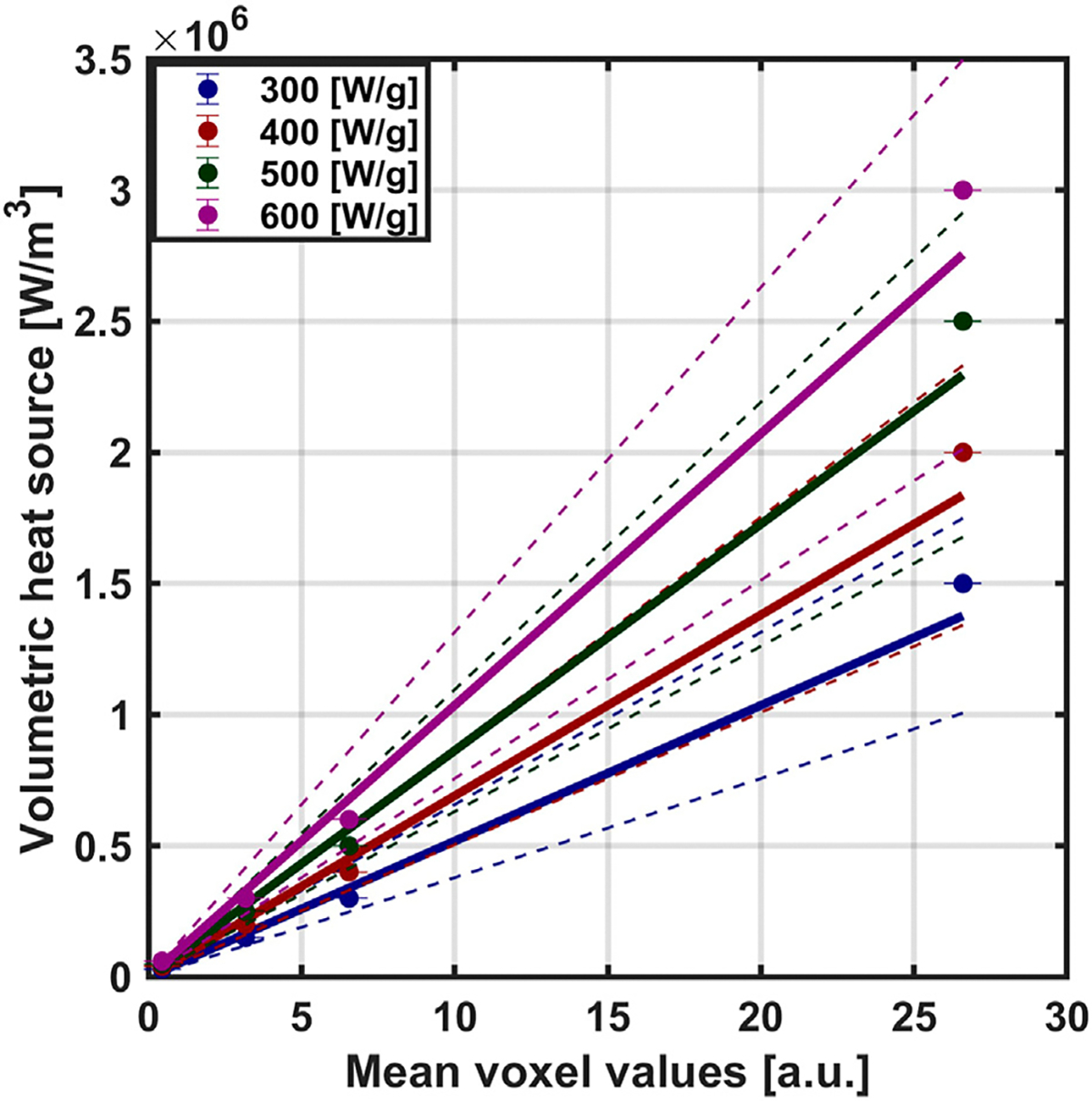
Converting MPI voxel intensity to volumetric heat output. Based on the MPI calibration samples, we first established a relationship between mean MPI signal and concentration. After multiplying concentration by SLP, the relationship provides a useful conversion between MPI signal and volumetric heat output, which we use in our simulations. The calibration curve was obtained by weighted least square regression with slopes [MW × (m^3^ × a. u.)^−1^] were fixed at the origin: 0.052 for 300 [W/g Fe]; 0.069 for 400 [W/g Fe]; 0.086 for 500 [W/g Fe]; and 0.104 for 600 [W/g Fe]. The solid line represents the regression fit with dotted line representing the 95 [%] confidence interval.

Using our MION MPI calibration data (shown in Supplementary Figure S1 in Supplementary Material), we created calibration curves for each SLP value, which yielded linear relationships between volumetric heat generation and MPI mean voxel value from weighted least square regression. The fitted slopes [MW × (m^3^ × a. u.)^−1^] were fixed at the origin: 0.052 for 300 [W/g Fe]; 0.069 for 400 [W/g Fe]; 0.086 for 500 [W/g Fe]; and 0.104 for 600 [W/g Fe] (Figure 2). Linear relationships were observed for all SLP values.

### 3.2 Simulated temperature rise

**FIGURE 3:**
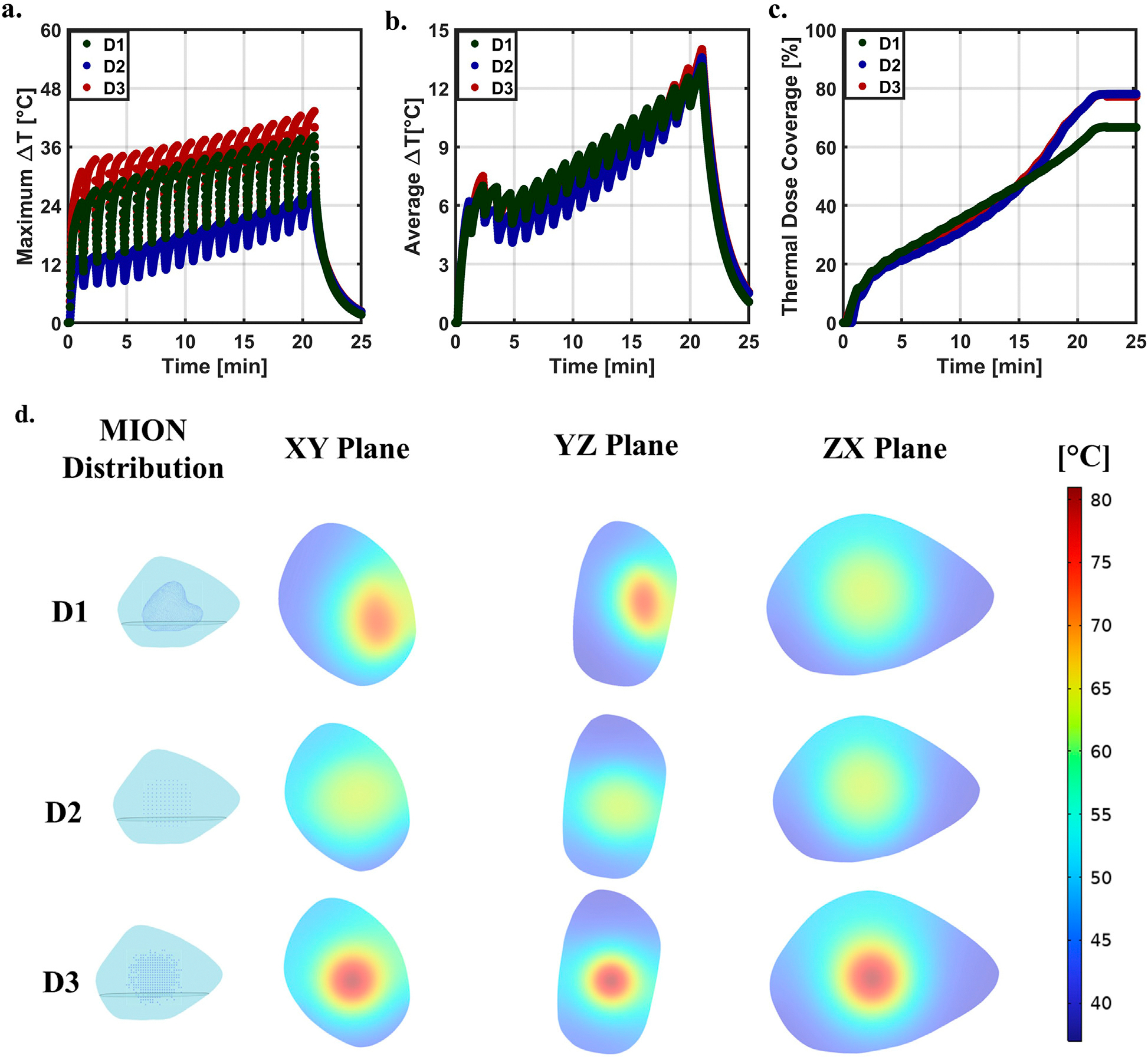
Distribution comparison at SLP of 600 [W/g Fe] and LVD of 4.06 [voxel/mm] [66.92 (voxels/mm^3^)] across the three distributions. **(A)** Predicted *maximum ΔT* for distributions D1, D2, and D3 as shown in Figure 1A. **(B)** Predicted *average ΔT* for three distributions. **(C)** Predicted *thermal dose coverage* for distributions D1, D2, and D3, respectively. **(D)** Temperature distribution at the end of the heating cycle of 21 [min] in the three planes (*xy*, *yz*, and *xz*) for the three distributions at an SLP of 600 [W/g Fe] and LVD of 4.06 [voxel/mm] [66.92 (voxel/mm^3^)].

For our thermal simulations, notable differences among the MION distributions emerged. With regard to *average* ΔT (Figure 3A), the D3 MION distribution had the highest temperature, followed by D1 and D2; accumulated thermal dose and associated dampening of perfusion-based convection resulted in a gradual rise over time. D2 led to a greater volume of tumor damage; thus, causing an increase in the rate of temperature change after 13 (min). The *maximum* ΔT (Figure 3B) was greatest for the D3 MION distribution, followed by D1 and D2, respectively, due to high localized heating at the center of the tumor.

Thermal dose (Figure 3C) differed slightly among the three test cases. Initially, D1 showed the greatest volumetric thermal dose, followed by D3 and D2, respectively; however, by the end of the simulated treatment time, D1 had the lowest accumulated thermal dose with D3 and D2 having approximately the same thermal dose. Inhomogeneous distributions of MIONs within the tumor produced insufficient thermal dose in regions containing fewer MIONs. D3 initially displayed a higher thermal dose because of the higher temperature at the tumor center; however, the D2 generated a more uniform temperature, leading to a higher coverage index than that of the D3.

By the end of simulated heating [21 (min)], temperature distributions across the phantom tumors and MIONs in three planes (*xy*, *yz*, and *xz*) varied owing to tumor and MION distribution inhomogeneities (Figure 3D). D2 and D3 showed a maximum temperature at the center of the tumor, which symmetrically decreased radially outward. However, D1 developed the highest maximum temperature with an offset to the center, as depicted in the *xy* and *yz* planes of the D1.

### 3.3 Effect of LVD on temperature and thermal dose

**FIGURE 4:**
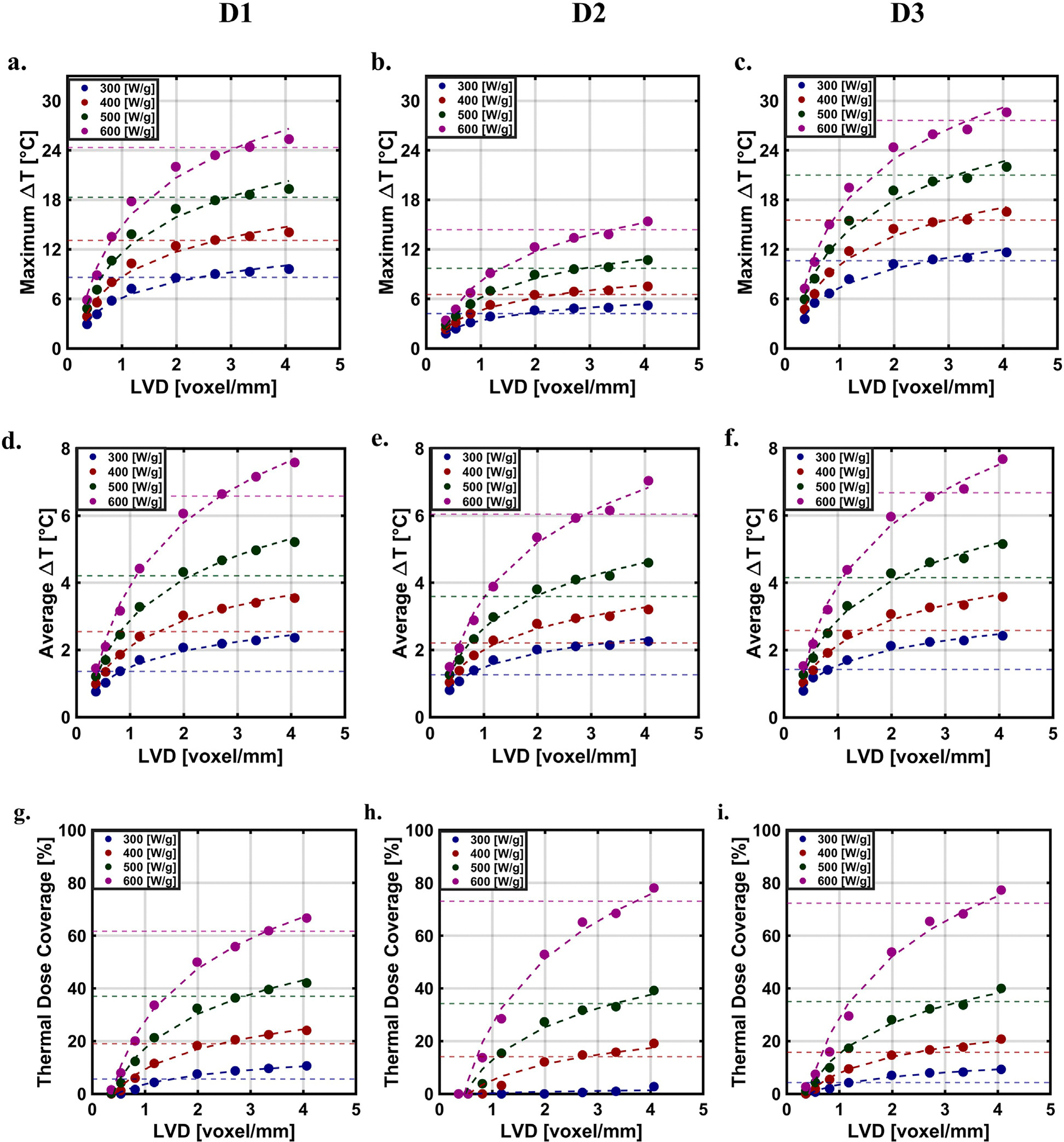
Simulated temperatures and thermal dose as a function of LVD with a logarithmic fit at different SLP’s across distribution. Predicted *maximum ΔT* **(A)** D1 **(B)** D2 **(C)** D3. Predicted *average ΔT* **(D)** D1 **(E)** D2 **(F)** D3. Predicted thermal dose. **(G)** D1 **(H)** D2 **(I)** D3.

The simulated *maximum* (Figures 4A–C) and *average* (Figures 4D–F) *ΔT* increased with LVD across all distributions. With decreasing resolution, spatial information about the MION distribution was lost. Areas containing high concentration of MIONs (high temperature) blended with nearby voxels occupied by lower concentrations of MIONs voxels as the voxel mesh was coarsened. Taken to its extreme, the distribution would converge to a single square voxel with an intermediate intensity and temperature.

The *thermal dose coverage* was lowest for the D1 distribution, which we attributed to its relatively higher heterogeneity, compared to those generated numerically (Figures 4G–I). MIONs in the D1 distribution appeared to be concentrated in the upper half of the XZ plane. For all distributions studied and for values of SLP less than 370 [W/g Fe], the coverage index approached convergence across all the distributions; however, when SLP values rose above 370 [W/g Fe], tumor coverage indexes failed to converge (Figures 4G–I). The thermal dose (CEM43) is exponential with respect to temperature; thus, a small change in the predicted temperature yields an exponential change in the thermal dose, producing a significant effect on coverage index.

### 3.4 Minimum spatial resolution and extrapolation

**FIGURE 5:**
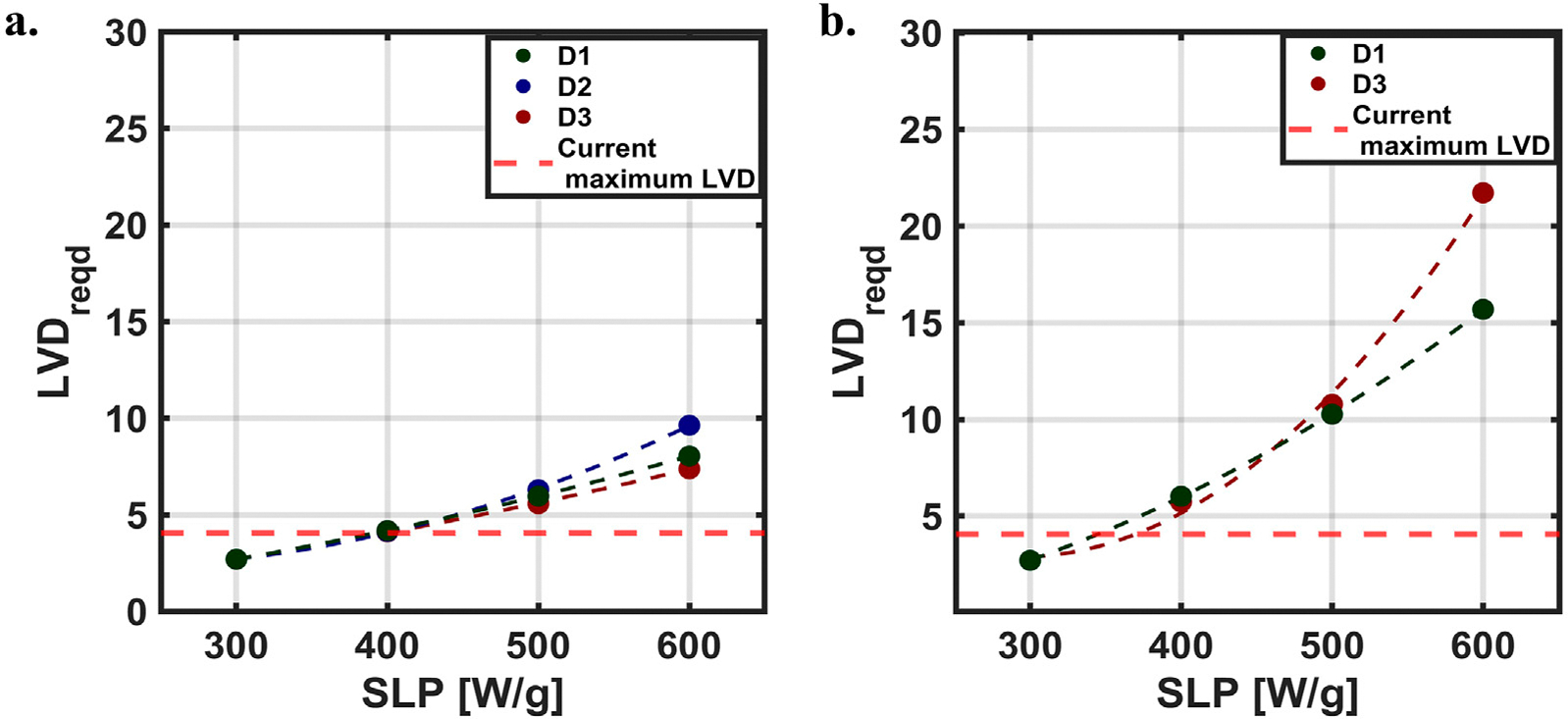
Required LVD ($LVD_{reqd}$) for simulation convergence. Relationship between $LVD_{reqd}$ and SLP, where the maximum LVD with our current MPI scanner is shown with the red dashed line. **(A)** *Maximum ΔT* **(B)** *Thermal dose coverage*.

LVD values at which the *simulated temperature* and *thermal dose coverage* exceeded our selection criteria [±1 (°C) and ±5 (%)], decreased with SLP. For example, when SLP = 300 [W/g Fe], the ±1 [°C] threshold was exceeded at 2.84 [voxel/mm], whereas for SLP = 600 [W/g Fe], this occurred at LVD = 3.60 [voxel/mm]. Extrapolating the logarithmic fits illustrates how particle SLP can affect $LVD_{reqd}$, i.e., the LVD required to achieve convergence in thermal simulations (Figure 5). Here, we assumed that LVD was saturated at $LVD_{\mathrm{sat}}=2.71$ [voxel/mm] for 300 [W/g Fe]. Quadratic interpolation $LVD_{reqd}$ (Figure 5A) revealed that particles having SLP values lower than 400 [W/g Fe] were able to achieve temperature convergence within the resolution limits of the Momentum MPI scanner used in this study [LVD = 4.06 (voxel/mm)].

The predicted values of *thermal dose coverage* indicate that a higher resolution would be needed to achieve convergence (Figure 5B), which arises from the exponential relationship between thermal dose and temperature. Results obtained from a quadratic interpolation, predicted that simulations conducted using SLP values between 300 and ~370 [W/g Fe] would achieve convergence within the capabilities of the MPI scanner LVD used for this study. The *thermal dose coverage* for D2 required to achieve convergence was ~0 when with SLP = 300 [W/g Fe] (Figure 4H). This resulted in *A*_*sat*_ ~0. For that reason, we discontinued further analysis of $LVD_{reqd}$ (Figure 5B) for D2. We concluded that *thermal dose coverage* for this diffused heat source was ~0 because an SLP of 300 [W/g] (or less) was insufficient to generate sufficient energy to maintain a temperature gradient that would manifest a significant thermal dose over the volume of tumor. On the other hand, the predicted minimum resolution required to achieve *thermal dose coverage* of ± 5 [%] for the other MION distributions depended on MION heat output (SLP). Minimum required spatial resolution to achieve convergence for D1 was $LVD_{reqd}=7,\;11$ or 16; and for D2, $LVD_{reqd}=7,\;11$ or 23 [voxel/mm] with MION SLP = 400, 500 or 600 [W/g Fe], respectively.

## 4 Discussion

MNPH offers many advantages in thermal medicine because the heat source (s) (nanoparticles) can be embedded in the target tissue and remotely activated. The nonlinear hysteresis response of some magnetic iron oxide nanoparticle (MION) formulations that generate heat in response to AMF enables control of the energy deposited in the tumor with amplitude adjustments. Challenges associated with MNPH arise from the dependence on MION concentration and distribution in tissue, and on the biological response (s) to heat, which depends on the duration of exposure to temperature. Time of exposure at temperature defines the thermal dose, and the thermal dose achieved in the tumor versus the surrounding tissues determines the therapeutic ratio.

Unpredictable (and uncontrollable) distributions and concentrations of MION deposits in tumors occur during and after delivery. As MIONs are a source of heat, their concentration in the tissue, and to a lesser extent, distribution in the tumor defines how much energy can be deposited. AMF amplitude adjustments during treatment can directly influence heating and tissue temperature rise. Thus, the operator can control energy deposition (provided sufficient MION content) and the temperature achieved in tissue. The ability to control energy deposition so that it conforms to a prescribed treatment plan requires knowledge of MION content and distribution in tissue, accurate thermometry in real time during treatment, and a treatment plan that accurately models tissue response to energy inputs. Other energy-based interventions such as radiation therapy require prescriptive treatment plans to ensure adequate patient safety and quality assurance of treatments. These are generated by computational models anchored on anatomical imaging data.

MPI can provide information of MION distribution and content in tissues without interference or ambiguities arising from anatomical features and variation, such as tissue density, composition (e.g., bone, fat, muscle), or air-filled volumes (e.g., lungs). MPI data, however, must be co-registered with anatomical imaging data provided by another modality to enable treatment planning. Co-registration of MPI images could lead to errors/uncertainties that need to be eliminated or reduced as much as possible. Co-registration of the MPI and CBCT images ultimately relies on manual selection and alignment of the fiducials in 3D space, which depends on operator judgement and skill. Variations in resolution between the MPI and anatomical images could lead to uncertainty in the transformation as well. MPI on the human scale could introduce additional motion artifacts, when compared to small animal imaging, since imaging of humans would likely not involve immobilization by anesthesia.

Assuming that the computational model used to develop the treatment plan can accurately capture essential biological and physical processes associated with MNPH, the simulations must ensure that energy is conserved across all potential MION distributions encountered. Average ΔT can be used as a surrogate to provide reasonable estimation to ensure energy conservation as energy absorbed by tissue will produce a temperature change. Values of average *ΔT*° calculated using the highest resolution tested here [LVD = 4.06 (mm/voxel)] were Δ*T*° = 7.58, 7.03, and 7.63 [°C] for MION distributions D1, D2 and D3, respectively. Small changes in average *ΔT* can be attributed to convective losses resulting from non-linear perfusion and uneven convective losses from the boundary.

The resolution of MPI data defines both the reliability of the predictions and the computational demands required to complete the computations. Sensitivity analyses can aid in predicting uncertainty. In the present context, sensitivity analysis can increase confidence in predictions obtained from the computational models by exploring how the predicted values depend on relevant biological phenomena, such as blood perfusion and thermophysical parameters that can vary substantially. Some of these input parameters can also vary during treatment, which further limits accuracy of treatment planning. Sensitivity analyses of many parameters have been described for hyperthermia treatment; however, the effect of image resolution quality in hyperthermia treatment planning has received much less attention because direct thermal dosimetry remains a practical challenge to implement in clinic, thus most current clinical hyperthermia technologies are based on tissue energy absorption.

It is unsurprising that requirements for MPI voxel resolution depend on MION SLP to enable precise tissue heating predictions for MNPH. Low values of SLP produce less heating, which requires a lower degree of computational fidelity to calculate temperature gradients in small volumes of a phantom tissue. Blood perfusion further decreases temperature gradients, reducing demands on computing resources to account for sharp temperature changes in small tissue volumes, thus relaxing constraints on minimum voxel resolution to accurately predict temperature changes. On the other hand, practitioners using MIONs having low SLP may attempt to compensate by increasing the dose of MIONs delivered. Higher tissue concentrations of MIONs can introduce additional complexities by increasing potential for MPI signal saturation and leakage of MIONs out of the tumor. The former reduces MPI accuracy in regions containing high MION concentrations, whereas the latter imposes new demands on MPI spatial accuracy outside the target volume and raises risks of off target heating and damage to normal tissues.

Our results and analysis predict that MIONs having high SLP will impose requirements for greater spatial/voxel resolution from MPI data, especially in regions containing higher concentrations of MIONs because these regions will manifest sharp temperature gradients. For this reason, both MPI and computational modeling require higher spatial accuracy (density) to account for the higher energy density, i.e., Watts per unit volume of tissue, to ensure accurate temperature predictions. Accurate temperature prediction is essential for reliable MNPH treatment planning because reliability of treatments depends on achieving a faithful match of delivered dose to that prescribed. For thermal therapies, the dose is defined as duration of exposure to a defined temperature. Given the exponential relationship between temperature and thermal dose, a small deviation in the temperature substantially affects the total thermal dose. As results indicate, dosage in MNPH varies upon the distribution assumed for the computational simulations. Heterogenous deposition of thermal dose, such as that observed *in vivo* (D1) are expected to generate a lower coverage index; whereas, distributions displaying more homogenous and tumor-centered MION content, yielded a higher coverage index. In the present study for instance, MIONs in D1 had lower CI as they were more concentrated on top of the *XZ* plane of the tumor, whereas homogenous distributions D2 and D3 yielded lower values of CI.

Though higher concentrations of MIONs may not be required in cases, the inevitable heterogeneity of MION distribution in tissue may yield regions that saturate MPI signal. These areas are likely to manifest as “hotspots” that ablate tissue and raise risks of “runaway” heating that increase toxicity. Current small animal preclinical scanners lack both spatial resolution and dynamic range to accurately image MIONs at the concentrations used for MNPH, and to accurately predict tissue heating in small volumes, especially for MIONs having SLP values exceeding 370 [W/g Fe].

Ultimately, the two main sources that define MPI resolution are the maximum magnetic field gradient (s) achieved by the scanner, and the magnetic properties of the MIONs. Small animal pre-clinical scanners currently achieve high gradient fields [>5 (T/m)] because the distance from the magnet to the sample is short, enabling higher spatial resolution (sub-millimeter range). Nanoparticles that generate a high intensity signal with a point spread function (PSF) width of 5–10 [mT] are needed to realize this potential resolution. As the size of scanners increases for clinical applications, the same gradient field (s) at the sample (patient) may prove impractical or unachievable, reducing spatial resolution. With a bore size large enough to accommodate a human head, the maximum achievable gradient field using the same magnet technology used in small animal scanners would be reduced by an order of magnitude (~0.1–0.5 T/m) (Graeser et al., 2019). Assuming particles with ideal MPI properties [PSF < 10 (mT)], a clinical scanner would provide centimeter scale resolution. One might assume that the ensuing loss of spatial resolution accompanying a transition to clinical size scanners would reduce accuracy of MNPH treatment planning simulations; however, our data show that precision from simulations depends on the combination of MION heat output (SLP) and spatial resolution of imaging data. Reasonable precision of temperature predictions obtained from MNPH simulations can be achieved from data obtained from MPI scanners having moderate resolution provided SLP is within a similarly moderate range.

More to the point, the effect of imaging data resolution on the precision of MNPH simulations depends on both nanoparticle distribution in tissue and their heat output. For non-uniform distributions (D1 and D3), our results show that MIONs displaying intermediate values of SLP [~100–300 (W/g Fe)] within clinically safe AMF parameters {Atkinson criteria: *H* × *f* ≤ *4.85*×*10*^*8*^ [A/(m × s)]} were adequate to achieve the imposed criteria (Atkinson et al., 1984). On the other hand, simulations using a uniform MION distribution failed to predict adequate thermal dose, except when MION SLP was high [>300 (W/g Fe)]. Assuming a uniform MION distribution in simulations of MNPH has been a common practice. Results obtained from such simulations therefore must be interpreted carefully because they overestimate requirements of minimum SLP, and evidence shows such uniformity of MION distributions in tissues is likely unrealistic (Attaluri et al., 2011a; Attaluri et al., 2011b). Uniform MION distributions ought to be used sparingly in simulations of MNPH.

## 5 Summary and conclusion

The reliability of computational predictions of tissue temperature arising from MNPH have historically been a challenge because conventional clinical imaging modalities provide limited information on MION location and content in tissue to anchor models. MPI provides a new capability with potential to improve MNPH by enabling more reliable treatment plans. The reliability of tissue temperature predictions depends on the spatial resolution defined by the imaging data used as the input. An increased resolution is expected to enhance the reliability of predictions, but the increased resolution also increases the demand for data storage and computational resources. To determine the limits of spatial resolution needed to balance computational demands with reliable temperature predictions, we conducted a sensitivity analysis of the effect of voxel size on predicted temperature and thermal dose using a phantom tumor and MIONs based on an experimental syngeneic subcutaneous murine tumor model. We determined that spatial resolution requirements depended on MION heating output or SLP. MIONs having lower values of SLP required a minimum spatial resolution of 2.71 [voxel/mm] [19.90 (voxel/mm^3^)]. Simulations having lower resolution underestimated temperature predictions. However, MIONs having higher SLP required values of spatial resolution exceeding capabilities of current small animal preclinical MPI scanners to predict temperature and thermal dose within the imposed criteria. Study results generally aligned with expectations, but a surprising finding was that a uniform distribution (D1) amplified the resolution needed to predict the temperature within ±1 [°C] and raised minimum SLP needed to provide meaningful thermal dose coverage. We note that many *in silico* studies of MNPH, used to guide MION development, have assumed a uniform distribution of heat sources in phantom tumors. The future clinical application of MPI technology will enhance understanding of its capabilities and limitations, refining its role in guiding MNPH. Efforts should address the challenges of MNPH optimization, including balancing MION heat production (SLP), delivery and dosing, MPI scanner resolution, computational demands, and clinical constraints, to develop a practical workflow using diverse tumor models. Studies must systematically examine how input parameter uncertainty affects the credibility of image-based simulations.

## Supplementary Material

Suppl

## Figures and Tables

**TABLE 1:** Geometric and mesh parameters used in this study, as shown in Figure 1C.

Parameters	Values
Muscle Height	12.6 [mm]
Muscle Width	25.2 [mm]
Muscle Depth	25.2 [mm]
MION volume	149.14 [mm^3^]
D1 and D2 MION radius	3.3 [mm]
Total time	25 [min]
Step size	1 [s]
Mesh	Adaptive mesh (Tetrahedral from 27,376 to 62,498)
Initial Tissue Temperature	37 [°C]

**TABLE 2:** Material properties used for simulation in FEBHT Software (Banura et al., 2016).

Material properties^a^	Muscle^a^	Tumor^a^
Density	1,090 [kg/m^3^]	1,045 [kg/m^3^]
Specific heat	3,421 [J/kg K]	3,760 [J/kg K]
Thermal conductivity	0.49 [W/m K]	0.51 [W/m K]
Blood perfusion	0.003 [1/s]	0.0095 [1/s]
Metabolic heat source	6,374.5 [W/m^3^]	31,873 [W/m^3^]

a Material properties used in the simulations were obtained from (Banura et al., 2016).

## Data Availability

The raw data supporting the conclusions of this article will be made available by the authors, without undue reservation.
