# Supplementary material for: Magnetic particle imaging resolution needed for magnetic hyperthermia treatment planning: a sensitivity analysis
Source: Front Therm Eng. Author manuscript; Available in PMC 2026 Feb 16. (PMC12037136; doi:10.3389/fther.2025.1520951)
Supplement: Suppl [file NIHMS2071909-supplement-Suppl.pdf]

Magnetic Particle Imaging Resolution needed for Magnetic Hyperthermia Treatment Planning: A Sensitivity Analysis

Shreeniket Pawar [1,‡], Nageshwar Arepally [1,‡], Hayden Carlton [2], Joshua Vanname [1], Robert Ivkov [2-5,*†] and Anilchandra Attaluri [1,†]

1. Department of Mechanical Engineering, School of Science, Engineering, and Technology, The Pennsylvania State University Harrisburg, Harrisburg, PA 17057, USA
2. Department of Radiation Oncology and Molecular Radiation Sciences, The Johns Hopkins University School of Medicine, Baltimore, MD 21231, USA
3. Department of Oncology, Johns Hopkins University School of Medicine, Baltimore, MD 21287, USA
4. Department of Mechanical Engineering, Whiting School of Engineering, Johns Hopkins University, Baltimore, MD 21218, USA
5. Department of Materials Science and Engineering, Whiting School of Engineering, Johns Hopkins University, Baltimore, MD 21218, USA

* Correspondence: rivkov1@jhmi.edu (R.I.)

‡ Equal contributions: Joint first authors

† Equal contributions: Joint senior authors.

Supplementary Information:

Verification and Validation of the COMSOL Multiphysics solver for Magnetic Nanoparticle Hyperthermia:

The solver was verified and validated against the analytical and experimental solution given by Goldenberg et. al. and Andra et. al. [1,2]. The results show that the numerical solution agrees with the analytical and experimental results.

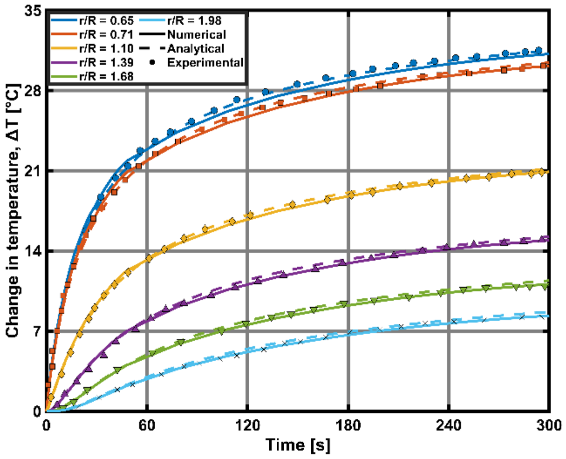

Figure S 1: Comparison between numerical, analytical and experimental results. The COMSOL Multiphysics yields results that agree to the experimental and analytical solution.

Calibration Curve for the Volumetric Heat Source from Magnetic Field:

The calibration curve for the volumetric heat source obtained from the magnetic field, mass of MION, MPI imaging and concentration of MION is shown in Figure S 2.

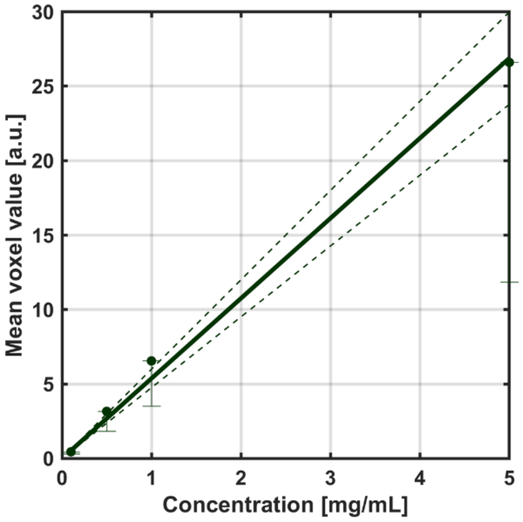

Figure S 2: a. Calibration curve for the mean voxel value obtained from concentration of MION from the MPI scanner.

Performance Metrics:

The performance metrics for the sensitivity analysis of the voxel resolution for the three distributions, that is, uniform, Gaussian, and *in vivo*, for the SLP's 300-600 [W/g] are listed in Table S 1.

Table S 1: Metrics for the sensitivity analysis of voxel resolution

			**LVD [voxel/mm]**							
			**4.06**	**3.34**	**2.71**	**1.99**	**1.18**	**0.81**	**0.54**	**0.36**
**300 [W/g]**	**D1**	**A**	0.75	1.02	1.37	1.70	2.07	2.18	2.28	2.36
		**B**	2.93	4.14	5.78	7.23	8.54	9.00	9.28	9.60
		**C**	3.59	5.15	7.79	10.02	12.13	12.81	13.26	13.34
		**D**	0.00	0.00	1.66	4.36	7.55	8.73	9.65	10.62
	**D2**	**A**	0.80	1.06	1.39	1.70	2.01	2.11	2.14	2.26
		**B**	1.80	2.39	3.15	3.87	4.62	4.85	4.93	5.21
		**C**	2.14	2.97	4.11	5.24	6.48	6.87	7.01	7.49
		**D**	0.00	0.00	0.00	0.00	0.00	0.53	0.96	2.76
	**D3**	**A**	0.79	1.18	1.41	1.70	2.12	2.24	2.28	2.42
		**B**	3.57	5.51	6.65	8.38	10.22	10.77	10.97	11.63
		**C**	4.38	7.10	8.77	11.33	13.99	14.78	15.06	15.99
		**D**	0.00	0.67	1.99	4.24	7.03	7.95	8.27	9.30
**400 [W/g]**	**D1**	**A**	0.98	1.34	1.86	2.40	3.03	3.23	3.40	3.54
		**B**	3.87	5.55	8.02	10.29	12.40	13.12	13.58	14.06
		**C**	4.90	7.44	11.29	14.78	18.02	19.06	19.77	20.45
		**D**	0.00	1.24	5.95	11.55	18.30	20.55	22.45	24.05
	**D2**	**A**	1.03	1.38	1.83	2.28	2.78	2.94	3.00	3.20
		**B**	2.32	3.13	4.20	5.27	6.48	6.87	7.01	7.50
		**C**	2.87	4.07	5.78	7.60	9.79	10.52	10.79	11.72
		**D**	0.00	0.00	0.00	3.18	12.13	14.75	15.80	19.15
	**D3**	**A**	1.03	1.40	1.92	2.45	3.07	3.26	3.34	3.58
		**B**	4.72	6.59	9.19	11.78	14.48	15.29	15.58	16.54
		**C**	5.99	8.68	12.52	16.20	19.98	21.12	21.53	22.88
		**D**	0.06	1.9	5.46	9.49	14.69	16.7	17.75	20.78
**500 [W/g]**	**D1**	**A**	1.21	1.69	2.45	3.28	4.32	4.66	4.96	5.21
		**B**	4.84	7.11	10.62	13.83	16.90	17.93	18.62	19.30
		**C**	6.30	9.87	14.87	20.05	24.64	26.10	27.17	28.12
		**D**	0.15	4.17	12.34	21.30	32.46	36.38	39.57	42.06
	**D2**	**A**	1.26	1.71	2.32	2.98	3.80	4.09	4.20	4.59
		**B**	2.85	3.90	5.37	6.96	8.93	9.59	9.84	10.7
		**C**	3.65	5.28	7.77	10.69	14.45	15.69	16.15	17.73
		**D**	0.00	0.00	3.86	15.40	27.31	31.73	33.07	39.22
	**D3**	**A**	1.27	1.77	2.50	3.31	4.28	4.60	4.72	5.15
		**B**	5.94	8.45	12.00	15.47	19.12	20.23	20.63	21.99
		**C**	7.73	11.43	16.51	21.38	26.54	28.15	28.74	30.75
		**D**	1.16	4.35	9.93	17.30	28.12	32.22	33.68	39.99
**600 [W/g]**	**D1**	**A**	1.45	2.09	3.16	4.42	6.06	6.64	7.15	7.58
		**B**	5.86	8.86	13.52	17.81	22.00	23.41	24.40	25.35
		**C**	7.84	12.24	19.61	25.83	31.94	33.94	35.47	36.04
		**D**	1.54	7.94	20.06	33.64	49.97	55.83	61.84	66.67
	**D2**	**A**	1.49	2.05	2.88	3.88	5.35	5.92	6.15	7.03
		**B**	3.39	4.72	6.73	9.11	12.27	13.39	13.81	15.39
		**C**	4.48	6.65	10.25	14.80	20.55	22.56	23.33	26.20
		**D**	0.00	0.04	13.73	28.49	52.88	65.12	68.45	78.07
	**D3**	**A**	1.53	2.18	3.20	4.38	5.96	6.55	6.78	7.67
		**B**	7.24	10.47	15.01	19.47	24.36	25.94	26.53	28.60
		**C**	9.27	13.83	20.02	26.20	33.39	35.80	36.72	40.02
		**D**	2.69	7.44	15.93	29.54	53.78	65.43	68.23	77.28

A: Average ΔT [°C] B: Maximum ΔT [°C] C: Maximum change in temperature at 1250 [s] (after heating cycle). D: Thermal dose at 1500 [s] (at end of the simulation).

Regression Parameter for the Logarithmic Curve Fitting:

Table S 2: Regression parameters obtained from the logarithmic fit.

	SLP [W/g]	Maximum ΔT [°C]		Average ΔT [°C]		Thermal dose [%]	
		**A**	**B**	**A**	**B**	**A**	**B**
**D1**	**300**	2.81	6.15	0.68	1.5	4.87	3.73
	**400**	4.34	8.7	1.1	2.12	10.83	9.47
	**500**	6.18	11.63	1.74	2.91	18.47	17.51
	**600**	8.33	14.93	2.67	3.95	28.23	27.95
**D2**	**300**	1.43	3.39	0.61	1.49	0.76	0.29
	**400**	2.18	4.64	0.91	2.01	8.74	5.27
	**500**	3.32	6.19	1.4	2.67	17.73	13.02
	**600**	5.07	8.2	2.31	3.59	35.32	26.79
**D3**	**300**	3.29	7.39	0.67	1.56	4.12	3.59
	**400**	5	10.14	1.08	2.16	8.72	8
	**500**	6.77	13.27	1.64	2.92	16.42	15.48
	**600**	8.96	16.77	2.58	3.94	33.07	29.22

Required Linear Resolution of the MPI Scanner

a. Linear regression:

Parameters obtained for the linear regression are enlisted in Table S 3 and shown in Figure S 3.

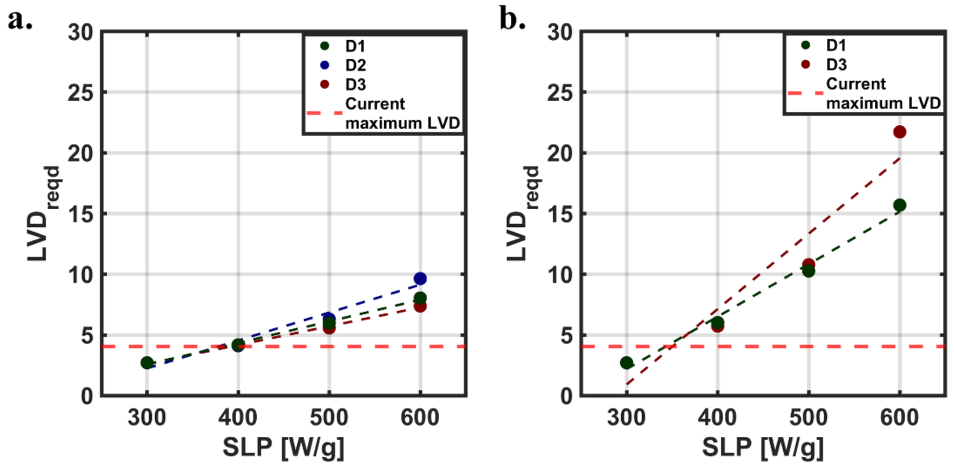

Figure S 3: Required linear voxel resolution from linear interpolation.

Table S 3: Linear regression parameters and SLP prediction for current SLP.

		Maximum ΔT [°C]	Average ΔT [°C]	Thermal dose [%]
D1	SLP [W/g]	384.3	370.53	343.21
	a [(voxel ×g^2) /(mm×W^2)]	0.02	0.03	0.04
	b[(voxel ×g) /(mm×W)]	-2.78	-5.63	-10.77
	R^2	0.99	0.97	0.99
D2	SLP [W/g]	378.7	379.21	-
	a [(voxel ×g^2) /(mm×W^2)]	0.02	0.02	-
	b[(voxel ×g) /(mm×W)]	-4.64	-5.42	-
	R^2	0.97	0.94	
D3	SLP [W/g]	392.74	371.61	350.52
	a [(voxel ×g^2) /(mm×W^2)]	0.02	0.03	0.06
	b[(voxel ×g) /(mm×W)]	-2.02	-5.46	-17.71
	R^2	1	0.97	0.92

b. Quadratic regression
   Parameters obtained for the linear regression are enlisted in Table S 4.

Table S 4: Quadratic regression parameters and SLP prediction for current SLP.

		Maximum ΔT [°C]	Average ΔT [°C]	Thermal dose [%]
D1	SLP [W/g]	391.98	390.03	345.02
	a [(voxel ×g^2) /(mm×W^2)]	0	5.30e-5	5.29e-5
	b[(voxel ×g) /(mm×W)]	0	-0.02	0
	c [voxel /mm]	0.07	4.61	-0.72
	R^2	1	1	1
D2	SLP [W/g]	399.45	408.69	-
	a [(voxel ×g^2) /(mm×W^2)]	4.79e-5	6.83e-5	-
	b[(voxel ×g) /(mm×W)]	-0.02	-0.04	-
	c [voxel /mm]	4.46	7.55	-
	R^2	1	1	-
D3	SLP [W/g]	399.17	387.33	369.92
	a [(voxel ×g^2) /(mm×W^2)]	1.00e-5	5.13e-5	1.98e-4
	b[(voxel ×g) /(mm×W)]	0.01	-0.02	-0.12
	c [voxel /mm]	-0.11	4.11	19.9
	R^2	1	1	1

Matlab Code

```
% Last Updated 12/2/2022 at 7:00 p.m.
% Reference Forum
% https://www.mathworks.com/matlabcentral/answers/892652-anybody-help-me-by-
providing-matlab-code-for-the-three-dimensional-histograms#answer_863405
% ---------------------------------------------------------------------------
------------------------------------------------------------------------------
clc; close all; clear all;
% ---------------------------------------------------------------------------
------------------------------------------------------------------------------
% Progress Bar for Script Completion
w = waitbar(0,'Please Wait...');
% ---------------------------------------------------------------------------
------------------------------------------------------------------------------
% Inputs
tic
% Import Data & Determine Voxel Count
data= readmatrix('Scaled_MNP.txt');
% ---------------------------------------------------------------------------
------------------------------------------------------------------------------
% Main Function

% 46 for Finsish to prevent points from being outside voxels
start=5;
finish=46; % 54 gives ~0.253 delta x on mesh


for iii=start:1:finish

    if iii==47 || iii==48
        continue
    end

% Max Number of Voxels along each axis
s=iii;

labelcolors = [0 0 .4; 0 .4 0; .4 0 0 ; 0 0 .6; 0 .6 0;.6 0 0;0 0 .8;0 0 .1;0 .1
0;.1 0 0;0 0 .3;
               0 .3 0;.3 0 0;0 0 .9;0 .9 0;0 .9 .9;.9 0 0;0 0 .5;0 .5 0;.5 0 0; 0
0 .2;0 .2 0;
               .2 0 0;0 0 1;0 1 0;0 1 1;1 0 0];  % must be rgb
x = data(:, 1);
y = data(:, 2);
z = data(:, 3);
label = data(:,4);
[groups, ID] = findgroups(label);
ngroups = length(ID);
if ngroups > size(labelcolors,1)
    error('too many groups, provide more labelcolors');
end
Nx = s; Ny = s; Nz = s;
xvox = (max(x)-min(x))/(Nx-1);
yvox = (max(y)-min(y))/(Ny-1);
zvox = (max(z)-min(z))/(Nz-1);
xidx = floor(x/xvox); xbin = xidx - min(xidx) + 1;
yidx = floor(y/yvox); ybin = yidx - min(yidx) + 1;
zidx = floor(z/zvox); zbin = zidx - min(zidx) + 1;
xq = xidx * xvox; yq = yidx * yvox; zq = zidx * zvox;
counts = accumarray([xbin(:), ybin(:), zbin(:)], 1, [Nx Ny Nz]);
```

```
occupied = counts > 0;
oind = find(occupied);
gcounts = cell(ngroups, 1);
for K = 1 : ngroups
    mask = groups == K;
    gcounts{K} = accumarray([ybin(mask), xbin(mask), zbin(mask)], 1, [Ny Nx Nz]);
end
gcounts4 = cat(4, gcounts{:});
[~, biggestgroupidx] = max(gcounts4, [], 4);
colidx = biggestgroupidx(occupied);
[ox, oy, oz] = ind2sub(size(occupied), oind);
oxq = (ox + min(xidx) - 1) * xvox;
oyq = (oy + min(yidx) - 1) * yvox;
ozq = (oz + min(zidx) - 1) * zvox;
noq = length(oxq);
rF = [1 2 3 4 1; 8 7 6 5 8; 1 4 6 7 1; 2 8 5 3 2; 1 7 8 2 1; 3 5 6 4 3];  %in
closed form
rV = [0 0 0; 1 0 0; 1 0 1; 0 0 1; 1 1 1; 0 1 1; 0 1 0; 1 1 0] .* [xvox, yvox,
zvox];
poxq = oxq(1:noq); poyq = oyq(1:noq); pozq = ozq(1:noq);
allF = repmat(rF(:,1:end-1), noq, 1) + 8*repelem((0:noq-1).', size(rF,1), 1);
allV = repelem([poxq, poyq, pozq], size(rV,1), 1) + repmat(rV, noq, 1);
[mappedV, ~, Vidx] = unique(allV, 'rows');
mappedF = Vidx(allF);
cdata = labelcolors(colidx,:);
% --------------------------------------------------------------------------------
--------------------------------------------------------------------------
% Plotting
% p = patch('Faces', mappedF, 'Vertices', mappedV, 'FaceVertexCData', cdata, ...
%     'LineWidth', 0.1, 'EdgeColor', 'none', 'FaceAlpha', 0.1);
F1=figure(1);
p = patch('Faces', allF, 'Vertices', allV, 'FaceVertexCData', cdata, ...
    'LineWidth', 0.1, 'EdgeColor', 'none', 'FaceAlpha', 0.1);
colormap(labelcolors);
xlabel('x'); ylabel('y'); zlabel('z');
view(3)
hold on
plot3(data(:,1),data(:,2),data(:,3),'.b')
hold off
axis square
movegui(F1,'west')
% % ------------------------------------------------------------------------------
----------------------------------------------------------------------------
% Find when Data is inside a Voxel and assign a Value to Voxel Verticies
vals_in=[];
count_in=1;
ind_in=[];
allV=[allV(:,:) zeros(length(allV(:,1)),1)];

for i=0:8:length(allV(:,1)) % 1 to number of voxels
if i==length(allV(:,1))
    % do nothing
else
xmin=min(min(allV(((i+1):(8+i)),1)));
xmax=max(max(allV(((i+1):(8+i)),1)));
ymin=min(min(allV(((i+1):(8+i)),2)));
ymax=max(max(allV(((i+1):(8+i)),2)));
zmin=min(min(allV(((i+1):(8+i)),3)));
zmax=max(max(allV(((i+1):(8+i)),3)));
```

```
    for ii=1:length(data(:,1))
        if (data(ii,1)>xmin & data(ii,1)<xmax) && (data(ii,2)>ymin &
data(ii,2)<ymax) && (data(ii,3)>zmin & data(ii,3)<zmax)
            vals_in(count_in)=data(ii,4);
            ind_in(count_in)=ii;
            count_in=count_in+1;
        else
        end
    end
% Convert to SLP (W/(m^3)) and divided by 8 for vertices in a voxel.
m_au=(26.124/26)*mean(vals_in)-1028.893231;
m_au = m_au-2;
m_slp=111349*m_au; % grey value to SLP

if m_slp < 0
    m_slp=0;
end

allV(((i+1):(8+i)),4)=m_slp/(1); % was divided by 8


% Prevent NaN values for voxel average
if isnan(mean(vals_in)) == 1
    error('NaN Detected!');
end
count_in=1;
vals_in=0;
end

end
% -------------------------------------------------------------------------------
-------------------------------------------------------------------------
% Output Text Files

% Update Progress Bar
F2=waitbar(iii/finish);


str1=num2str(iii);
C={'BME_504_TEXT_FILE_',str1,'.txt'};
str2=strjoin(C,'');
fileID = fopen(str2,'w');
writematrix(allV, str2,'Delimiter','space');
fclose(fileID);

end
% -------------------------------------------------------------------------------
-------------------------------------------------------------------------
% Close Progress Bar
close(w)
toc
```

References

1. Goldenberg, H., & Tranter, C. J. (1952). Heat flow in an infinite medium heated by a sphere. British journal of applied physics, 3(9), 296.
2. Andrä, W., d'Ambly, C. G., Hergt, R., Hilger, I., & Kaiser, W. A. (1999). Temperature distribution as function of time around a small spherical heat source of local magnetic hyperthermia. *Journal of Magnetism and Magnetic Materials*, *194*(1-3), 197-203.
